# Is delirium associated with pain and administered morphine in patients in the ICU after cardiac surgery?

**DOI:** 10.1186/cc9761

**Published:** 2011-03-11

**Authors:** L Van Gulik, H Brouwer, SJ Ahlers, W Van Boven, CA Knibbe, E Van Dongen, P Bruins

**Affiliations:** 1 St Antonius Hospital, Nieuwegein, the Netherlands; 2 University of Utrecht, the Netherlands

## Introduction

Delirium after cardiac surgery is associated with a prolonged length of stay in the ICU, prolonged ventilation time and higher in-hospital mortality. Although the exact pathophysiology of delirium is unknown, both the use of analgesics and the experience of pain have been suggested to be associated with the occurrence of delirium. The aim of the study was to evaluate the association between delirium and analgesics and pain in the ICU.

## Methods

In a retrospective observational study, pain and delirium scores in patients admitted to the ICU after cardiac surgery via sternotomy during a 2-month period were analyzed. Delirium was scored using the Intensive Care Delirium Screening Checklist (ICDSC, range 0 to 8, ≥4 was deemed delirious). Pain was scored on the Numeric Rating Scale (NRS, range 0 to 10, ≥4 was deemed unacceptable). Morphine was administered according to a pain titration protocol.

## Results

ICDSC ≥4 was recorded at least once for 32 (26%) of the 121 included patients. These patients received significantly less morphine than patients with all ICDSC scores <4 (mean dose 23 ± 8 mg/day vs. 29 ± 13 mg/day, *P *< 0.01), without difference in pain scores between the groups (mean NRS 1.3 vs. 1.4, *P *< 0.3 and 34% vs. 28%, *P *< 0.51 experienced at least one unacceptable pain score). Delirious patients were older (70 ± 9 vs. 66 ± 11 years, *P *< 0.03), and ventilation time and length of stay in the ICU were significantly longer (26 ± 34 vs. 14 ± 20 hours, *P *< 0.001 and 77 ± 53 vs. 48 ± 38 hours, *P *< 0.001 respectively). In-hospital mortality was significantly higher for this group (3 vs. 0 patients, *P *< 0.02).

## Conclusions

While delirious patients received significantly less morphine than nondelirious patients, there was no significant relation between delirium and pain in patients following cardiac surgery in the ICU.

